# Roles of multimodal intra-operative neurophysiological monitoring (IONM) in percutaneous endoscopic transforaminal lumbar interbody fusion: a case series of 113 patients

**DOI:** 10.1186/s12891-021-04824-2

**Published:** 2021-11-26

**Authors:** Yu Chen, Chunmei Luo, Juan Wang, Libangxi Liu, Bo Huang, Chang-Qing Li, Yue Zhou, Chencheng Feng

**Affiliations:** Department of Orthopaedics, Xinqiao Hospital, The Army Medical University, Chongqing, 400037 China

**Keywords:** Percutaneous endoscopic transforaminal lumbar interbody fusion, Multimodal intraoperative neurophysiological monitoring, Motor-evoked potentials, Somatosensory-evoked potentials, Electromyography

## Abstract

**Background:**

Despite the wide use of intraoperative neurophysiological monitoring (IONM) in spinal surgeries, the efficacy of IONM during percutaneous endoscopic transforaminal lumbar interbody fusion (PE-TLIF) surgery in detecting postoperative neurological deficits has not been well characterized.

**Methods:**

MIONM data from 113 consecutive patients who underwent PE-TLIF surgeries between June 2018 and April 2020 were retrospectively reviewed. Postoperative neurological deficits were documented and analyzed, and the efficacy and specificity of various IONM techniques were compared.

**Results:**

Of the 113 consecutive patients, 12 (10.6%) with IONM alerts were identified. The MIONM sensitivity and specificity were 100 and 96.2%, respectively. The frequency of neurological complications, including minor deficits, was 6.2% (*n* = 7); all of the neurological complications were temporary. The ability of single IONM modalities to detect neurological complications varied between 25.0 and 66.6%, whereas that of all modalities was 100%.

**Conclusions:**

MIONM is more effective and accurate than unimodal monitoring in assessing nerve root function during PE-TLIF surgeries, reducing both neurological complications and false-negative findings. We recommend MIONM in PE-TLIF surgeries.

## Background

Minimally invasive surgery (MIS) has been widely accepted as a better alternative for the treatment of lumbar spinal disorders [1–3]. Percutaneous endoscopic transforaminal lumbar interbody fusion (PE-TLIF) surgeries directly realize decompression and fusion without destruction of the posterior spinal components. However, due to the insufficient operative field and the limited exposure of anatomical landmarks, the risk of iatrogenic neurological injury is increased during PE-TLIF surgeries [4, 5].

To assess the online functional integrity of nerve roots during PE-TLIF, intra-operative neurophysiological monitoring (IONM) has been extensively used [6]. This procedure helps guide operative procedures, reduces neurological complications and improves surgical safety [7]. Currently, various IONM modalities have been applied in PE-TLIF [8, 9], including electromyography (EMG), somatosensory evoked potentials (SSEPs) and transcranial motor evoked potential (MEP), which provide real-time feedback to surgeons with information concerning potential nerve root insults during PE-TLIF.

Unimodal electromyography (EMG) is routinely used to monitor nerve root function during PE-TLIF [10], whereas EMG monitoring conveys difficulty in determining whether nerve function has been affected. Thus, the unimodal IONM method has some limitations. Therefore, multimodal IONM (MIONM) has been proposed as a novel IONM method.

EMG is not a test of neural integrity; therefore, detection of EMG in iatrogenic injury is severely limited [10, 11].Somatosensory evoked potentials (SSEPs) have been proven effective in monitoring spinal cord function during cervical and chest surgery, but there is still controversy regarding the monitoring of lumbar spine surgery [12–14]. Animal studies have shown that MEP is highly sensitive and specific for predicting injury [15–17], and clinical studies have shown that MEP monitoring effectively detects human nerve root injury during spinal deformity correction [18, 19]. Therefore, a multimodal IONM (MIONM) in PE-TLIF surgery may provide a more comprehensive assessment of neurological integrity. However, relevant evidence for this hypothesis is still insufficient.

We retrospectively analyzed waveforms of MEP, SSEPs and EMG from patients undergoing PE-TLIF procedures at our center and compared the sensitivity and specificity of these individual IONM modalities. Moreover, to standardize multimodal IONM procedures during PE-TLIF surgeries, the best combination of IONM modalities for detecting nerve root deficits intra-operatively was determined.

## Methods

### Study design

This study was conducted with the approval of the Institutional Review Board of Xinqiao Hospital. All patients included in this study signed informed consent form. Here in, we retrospectively reviewed a series of consecutive PE-TLIF patients seen at a single spine center between June 2018 and April 2020. All surgeries were performed by trained full-time orthopedic surgeons using MIONM. Inclusion criteria included patients with lumbar spinal stenosis, spondylolisthesis, and degenerative lumbosacral spine diseases with instability, radiculopathy, or neurogenic claudication that did not respond to conservative treatments and required unilateral neurologic decompression. Exclusion criteria included the presence of (1) serious underlying diseases or mental illnesses; (2) cauda equina syndrome or active infection;(3) previous lumbar surgical treatment, ozone intervention, or radiofrequency ablation; (4) bilateral neurologic decompression; (5) bleeding disorders, coagulation abnormalities, or pre-operative anemia; (6) unwillingness or inability to participate in treatment and complete follow-up; or (7) a related electronic device implant; (8) absent SEP or MEP waveforms, unilateral or bilateral.

### Anesthesia Protocolxd

General anesthesia was induced with a bolus dose of propofol (1–2 mg/kg), midazolam (0.03–0.05 mg/kg) and fentanyl (0.25 ~ 0.5 μg/kg) combined with a short-acting muscle relaxant, cisatracurium (0.15–0.2 mg/kg), and an inhalation agent (sevoflurane). No muscle relaxants or inhalation agents were administered after induction and intubation. Subsequently, anesthesia was maintained with propofol (3–6 mg/kg/h), based on haemodynamic response, and remifentanyl (0.15–0.3 μg/kg/min). The sedation depth monitoring index was observed using BIS/Narcotrend, and BIS values were maintained at 40–60. The train-of-four (TOF) twitch test was used to monitor metabolism, and the TOF ratio was maintained at values greater than 70%.

### Surgical technique

**Fig. 1:**
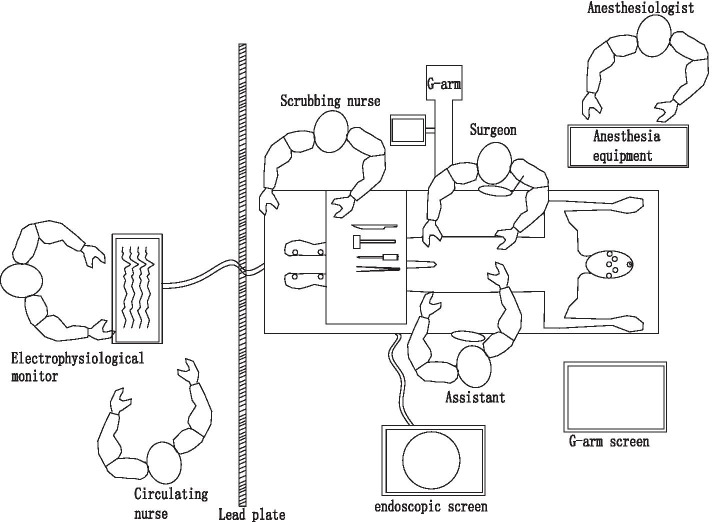
The setting of operation room during PE-TLIF

**Fig. 2:**
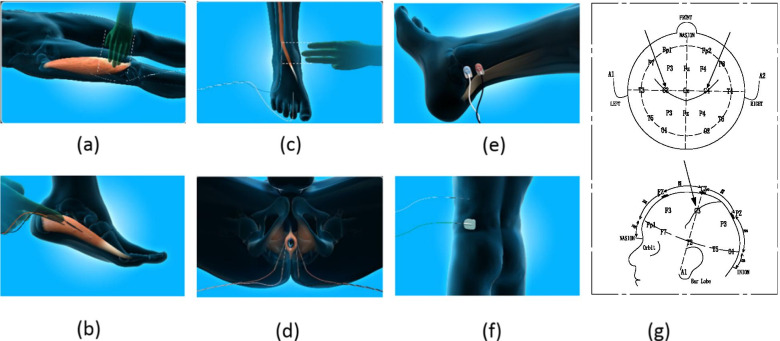
The electrode placement position during PE-TLIF. **a** Vastus lateralis; **b** Abductor pedis; **c** Extensor pollicis longus; **d** Anal sphincter; **e** Posterior tibial nerve; **f** ground connection; **g** Transcranial electrode placement

**Fig. 3:**
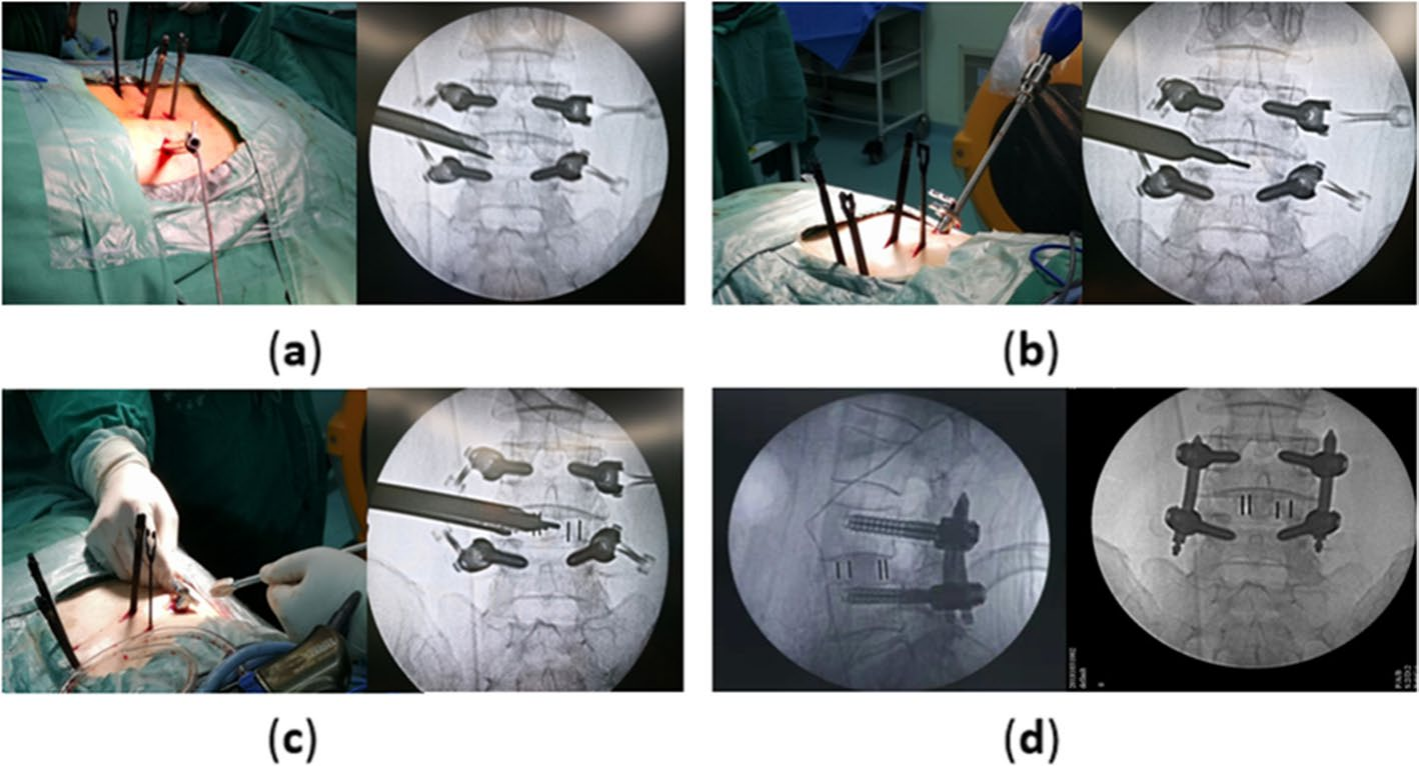
**a** Bilateral percutaneous pedicle screw instrumentation and the working tube placement; **b** Lumbar discectomy was performed under endoscopic spinal system; **C** A cage was implanted via a tail-end expandable tubular system; **d** Postoperative plain films of lumbar spine

The setting of operation room is shown in Fig. 1, The electrode placement position is shown in Fig. 2. After general anesthesia, with the patient prone on the operating table, electrode wires for IONM were quickly connected. First, bilateral percutaneous pedicle screw fixation was performed via a posterolateral Wiltse approach [3] at the responsibility levels. A routine PELD operation was performed [20]. Intra-operative fluoroscopy confirmed the location of the working cannula (Fig. 3a). Nerve root decompression and discectomy were performed (Fig. 3b).

Next, the TESSYS working canula was replaced with a tail-end expandable tubular dilator (PELIF®, Sanyou, Shanghai, China) [2], and the dilator was inserted into the intervertebral disc with a twisting movement through the caudal dilation tube using instruments, such as raspatories, pituitary rongeurs, and curettes, to prepare the endplate. Finally, under the monitoring of IONM, a cage (Halis®, PEEK material, Sanyou, Shanghai, China) was implanted (Fig. 3c). After the rod is set, the set screws were tightened. Figure 3d shows the postoperative plain film.

### Method and principles of MIONM

Using a 16-channel multi-function monitor, continuous and uninterrupted joint monitoring of MEP, EMG, and SSEPs was performed in different time phases. IONM test selection for each case was based on the surgeon’s request with the guidance of a neurologist consult.

Motor-evoked potential (MEP) was elicited using subcutaneous needle electrodes stimulating at a constant voltage (400–500 V) and multiple trains of 5 to 7 pulses with a duration of 200 to 400 ms for each pulse. The interstimulus interval was 2.0 to 4.0 ms for each stimulation train. The recording electrode was placed on the muscle innervated by the corresponding nerve root, and the compound muscle action potential caused by the stimulation was recorded.

Somatosensory evoked potential (SSEP) involved the stimulation electrodes being placed at the posterior tibial nerve (PTN) at the ankle. The stimulation intensity ranged from 35 to 45 mA with a stimulation rate of 2 Hz, and 160 to 300 trials were averaged for each trace. Responses were recorded in a referential fashion from multiple electrodes with fixed Cz and Fz(International 10–20 System). Primarily the P40 incubation period and amplitude of SSEP of both lower extremities were recorded.

Electromyography (EMG) monitoring is divided into triggered electromyography (Tr-EMG) and free electromyography (F-EMG). The former is discontinuous monitoring used to judge the integrity of the pedicle screw and identify adjacent nerve structures, while the latter continuously monitors EMG changes caused by nerve root traction, compression and manipulation stimulation, as well as pedicle screw placement damage.

### Warning criteria

MEP: The warning standard was that the waveform completely disappeared or the amplitude decreased by 80% from baseline [21].

SSEP: Continuous recording was compared to the baseline trajectory, and reductions in the amplitude by at least 50% or increases in the delay by 10% served as alarm criteria [22].

F-EMG: Explosive muscle contraction reaction occurs continuously, especially muscles dominated by nerve roots that might be damaged by surgery, serving as the warning standard. F-EMG activity was recorded using the same recording myotomes as for CM-EP responses. If one observed neurotonic discharges lasting longer than 5 s, this elicited a CM-EP trial [12].

### Neurological complication definition

Nervous system examinations were performed before and after surgery, including assessment of changes in limb muscle strength and sensation. A neurological complication was defined as any new neurological symptom and/or sign or worsening of pre-existing symptom and/or sign occurring immediately after surgery and having either a transient or permanent nature. The final clinical evaluation was performed by the neurologist.

### Data analysis

#### True-positive (TP)

A change in evoked potential (EP) followed by a new neurological disorder being observed during the wake-up test or at the end of surgery.

#### True-Negative (TN)

During the entire operation, compared to baseline values, the evoked potential changed within normal ranges, and no neurological deterioration was observed after surgery.

#### False-Negative (FN)

Throughout the surgery, the evoked potentials remained consistent with baseline values, but post-operative neurological examination indicated new neurological defects.

#### False-Positive (FP)

The evoked potential (EP) changed, resulting in corresponding measures being taken that did not eliminate the alarm, but there were no new neurological defects observed during the wake-up test and no new defects at the end of surgery.

#### Indeterminate

There was an alarm, the surgeon adjusted the surgical method, the alarm was eliminated, and there were no new neurological defects after surgery. However, it was difficult to determine whether this was because of the alarm after taking measures to avoid post-operative neurological defects.

- Sensitivity was defined as TP/ (TP+ FN) *100%.
- Specificity was defined as TN/ (TN + FP) *100%.
- Positive predictive value (PPV) was defined as TP/ (TP+ FP) *100%.
- Negative predictive value (NPV) was defined as TN/ (TN + FN) *100%.

## Results

### Patient population

Demographic and clinical data for all 113 patients is shown in Table 1. The male to female ratio was 1:0.89, the average age was 37.4 ± 7.8 years (range: 23–68 years), and the mean height and body mass index (BMI) were 169.7 ± 6.2 cm (range: 155–183 cm) and 17.9 ± 5.22 (range: 12–35), respectively. The average surgery time was 209.0 ± 29.1 min (range: 170–300 min), and intra-operative blood loss averaged 267.1 ± 77 ml (range: 100–500 ml). Out of 113 patients, surgical levels included L2-L3 (11.5%), L3-L4 (19.5%), L3-L5 (11.5%), L4-L5 (42.5%), and L5-S1 (15.0%) (Table 1).

**Table 1:** Demographic and clinical data of Patients (*n* = 113)

General data	
Male: female	1:0.89
Age, mean ± SD (range)	37.4 ± 7.8 (23–68 yr)
Height, mean ± SD (range)	169.7 ± 6.2 (155–183 cm)
Weight, mean ± SD (range)	67.4 ± 8.7 (47–90 kg)
BMI, mean ± SD (range)	17.9 ± 5.22 (12–35)
Operation time	209.0 ± 29.1(170-300 min)
Bleeding volume	276.1 ± 77.1(100-500 mL)
One vertebral level N (%)	100 (88.5%)
Two vertebral levels N (%)	13 (11.5%)
L2-L3 N (%)	13 (11.5%)
L3-L4 N (%)	29 (25.7%)
L4-L5 N (%)	54 (47.9%)
L5-S1 N (%)	17 (15.0%)

*BMI* indicates body mass index, *SD* standard deviation

### Neurological complications

**Fig. 4:**
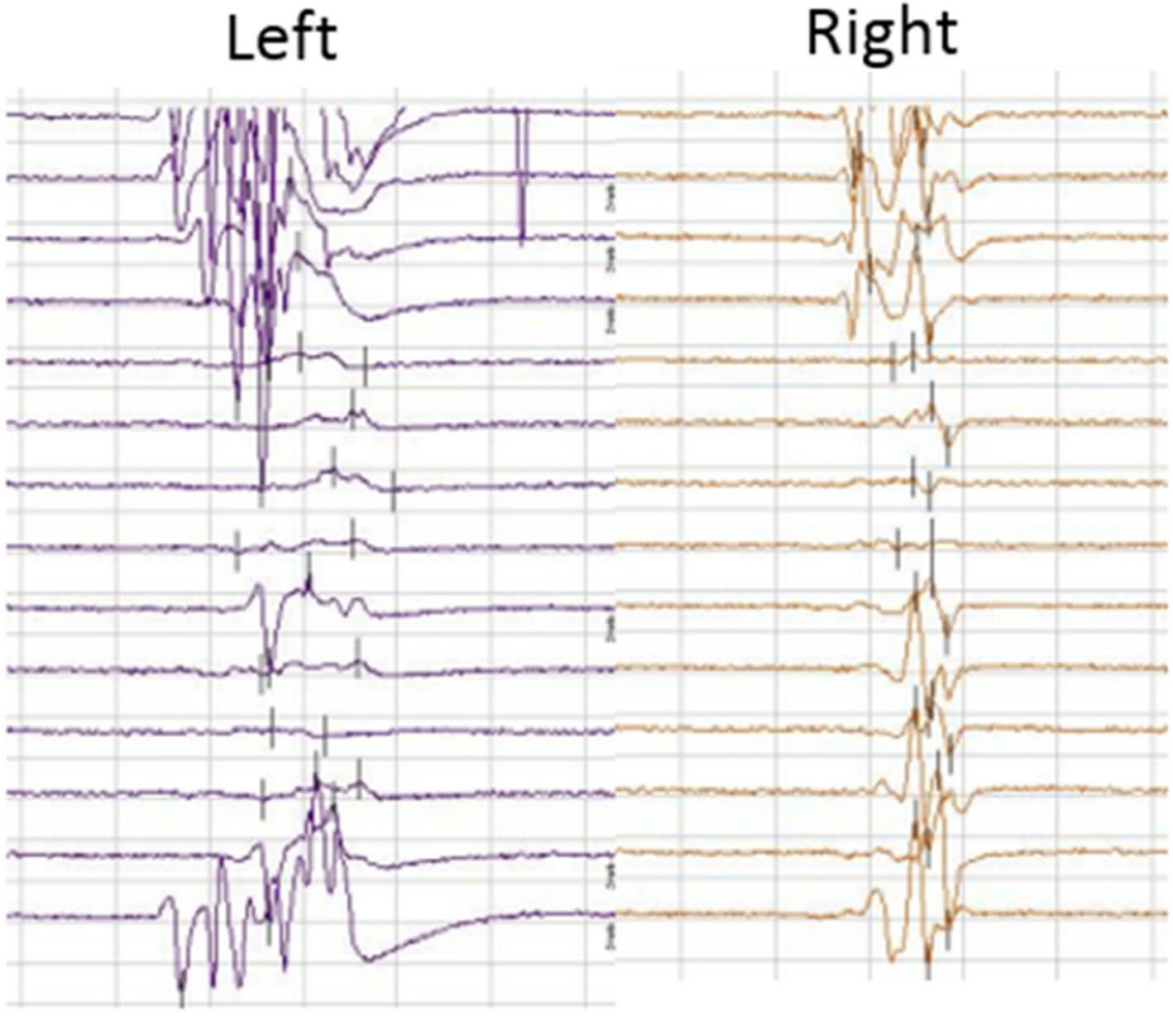
The typical motor-evoked potential (MEP) traces from a 38-year-old female with spondylolisthesis L5/S1 grade III. intraoperative MEP trace disappeared during the operation and appeared after treatment, recovery after surgery without neurological deficits

A total of 7 (6%) neurological complications were recorded during the post-operative period (Table 2). Out of 7 cases, 2 exhibited sensory deficits and pathological SSEP baselines pre-operatively [23], Both cases have the problem of prolonged latencies. All 7 cases presented with motor deficits post-operatively (2 cases showed right lower extremity weakness (3/5), 1 case showed left lower extremity weakness (4/5), and 3 cases showed bilateral muscle weakness (3/5)). Moreover, 2 cases complained of newly appeared sensory deficits post-operatively (1 case showed numbness of the left thigh and hip, while another experienced numbness in the left back portion of the feet). Fortunately, all neurological deficits were transient and minor, and these complications disappeared within 5–7 days after surgery. Figure 4 demonstrates the value of MIONM and its impact on the surgical procedure in one specific case of a 38-year-old female with spondylolisthesis L5/S1 grade III.

**Table 2:** List of patients’ IONM tests with postoperative neurological deficits (n = 7)

N	Region	Mainly monitored muscles	preoperative deficit	OP time	baseline	Test With EP Changes	Neurological deterioration	Recovery Time
1	L4-L5	Tibialis anterior	+	200 min	Pathological ncEP	MEP EMG	motor deficit sensory deficit	5 day
2	L3- L4	Rectus femoris	–	180 min	All potentials normal	MEP EMG	motor deficit	5 day
3	L3- L4	Rectus femoris	–	190 min	All potentials normal	MEP SEP	motor deficit	7 day
4	L5-S1	Gastrocnemius lateral head	–	210 min	All potentials norma	SEP MEP EMG	motor deficit sensory deficit	6 day
5	L5-S1	Gastrocnemius lateral head	+	170 min	Pathological ncEP	MEP EMG	motor deficit	5 day
6	L3- L5	Rectus femoris, Tibialis anterior	–	220 min	All potentials normal	SEP EMG	sensory deficit motor deficit	7 day
7	L4-L5	Tibialis anterior	–	160 min	All potentials norma	MEP SEP	motor deficit	7 day

*IONM* intra-operative neurophysiological monitoring, *OP* operation, *M* man, *F* faman, *EMG* electromyography, *SSEP* spino-spinal evoked potentials, *MEP* spino-muscular evoked potentials, *EP* evoked Potentia

### Intra-operative electrophysiological monitoring and treatment

Out of 113 patients, 12 cases showed intra-operative MEP changes above the alarm threshold. 6 of them were true positive, 4 of them were False positive. The MEP of two cases recovered after taking measures including stopping the operation, adjusting the cage or flushing with warm saline, etc. These two cases had no neurological deficits after surgery. Therefore, they were determined as indeterminate.

Eleven patients had intra-operative SSEP changes, 9 of whom did not develop post-operative neurological deficits and one of whom exhibited changes that after treatment, resulted in recovery and no change being observed after surgery. In contrast, out of these 11 patients, 2 of them experienced new neurological deficits after surgery. Furthermore, out of patients with post-operative neurological deficits, 5 showed no changes in SSEP tests.

**Fig. 5:**
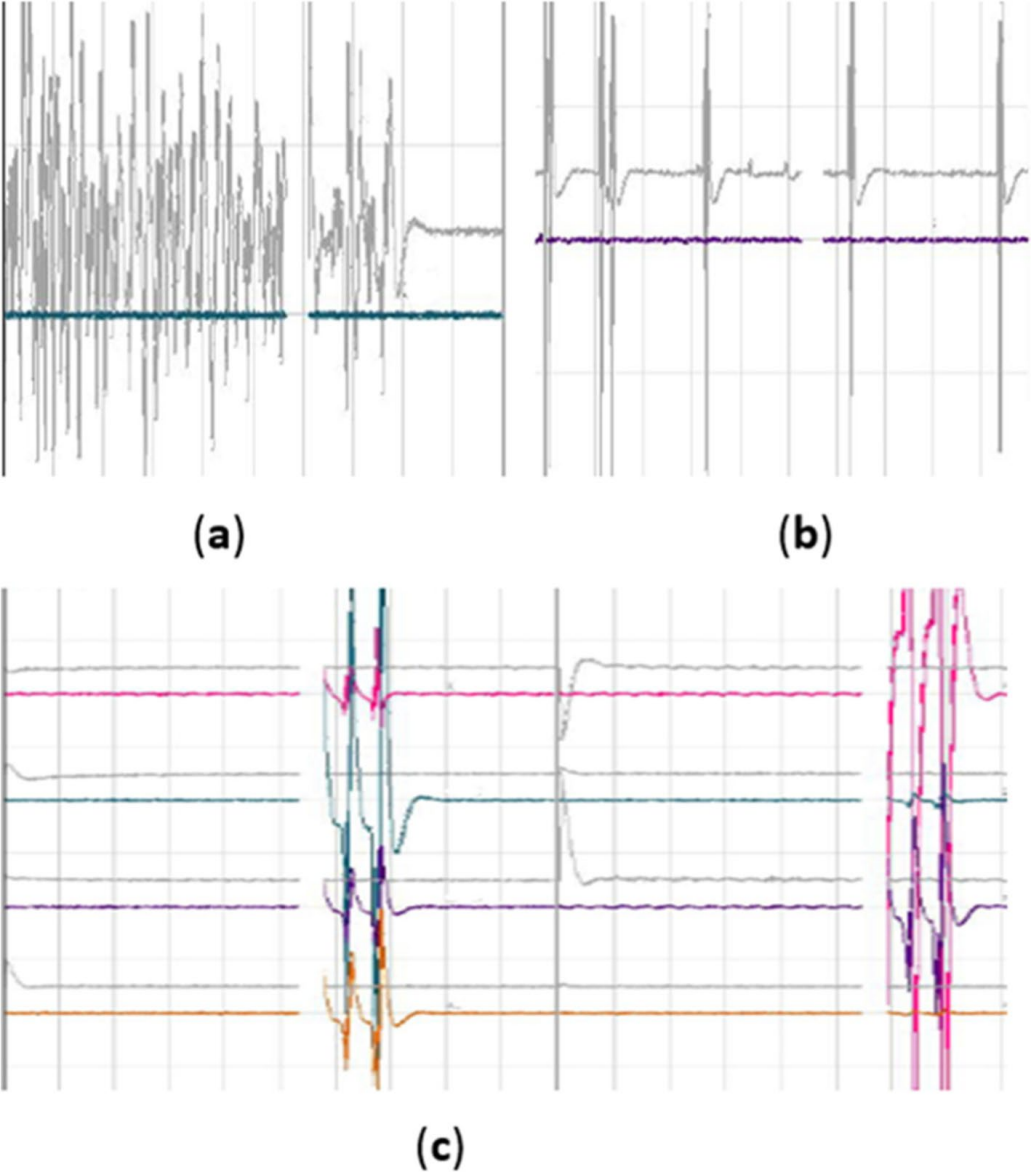
**a** Bursting activity caused by traction nerve. It was characterized by long duration and irregularity; **b** non-bursting regular activity caused by implanted surgical channel, It was characterized by a short duration and appears with the striking of the bone hammer; **C** interference waveform caused by bipolar radiofrequency burning, It was characterized by being messy and appears with the use of bipolar radiofrequency

With regard to EMG monitoring, the results showed that out of all 113 patients, 113 exhibited EMG activity, but most of them appeared during placed surgical access, and when surgery was paused, the activity immediately disappeared (Fig. 5. Eleven patients showed EMG activity, 5 of whom exhibited new neurological deficits after surgery, 4 of which were accompanied by MEP changes,1 accompanied by SSEP changes, and 1 accompanied by both SSEP and MEP changes.

### IONM specificity, sensitivity, and positive and negative predictive values

The sensitivity and specificity, respectively, of each modality of monitoring were as follows: SSEP only (28.5, 92.39%); MEP only (85.7,96.2%); EMG only (71.4, 94.3%); MEP and EMG (85.7, 97.1%); and multimodal IONM (100, 97.1%). The positive and negative predictive values, respectively, were as follows: SSEP only (20.0, 95.0%); MEP only (60.0, 99.0%); EMG only (45.4, 98.0%); MEP and EMG (66.7, 99.0%); and multimodal IONM (70, 100%) (Table 3).

**Table 3:** Value of unimodal and multimodal IONM techniques in detecting neurological complications during PE-TLIF (n = 113)

	Intraoperative monitoring techniques				
	EMG	MEP	SSEP	EMG/ MEP	EMG/ MEP /SSEP
True positive	5	6	2	6	7
True negative	100	100	97	101	101
False positive	6	4	8	3	3
False negative	2	1	5	1	0
Indeterminate	0	2	1	2	2
Sensitivity (%)	71.4	85.7	28.5	85.7	100
Specifcity (%)	94.3	96.2	92.3	97.1	97.1
Positive predictive value (%)	45.4	60.0	20.0	66.7	70
Negative predictive value (%)	98.0	99.0	95.0	99.0	100

*EMG* electromyography, *SSEP* spino-spinal evoked potentials, *MEP* spino-muscular evoked potentials

## Discussion

During general anesthesia, surgeons can’t monitor patient lower limb sensation and movement in real time, correspondingly increasing the potential risk of nerve damage. Post-operative causal nerve root pain and abnormal sensory movements of the lower extremities are the most common complications after percutaneous endoscopic surgery of the lumbar spine [24]. In our clinic, multimodal IONM (EMG + MEP + SSEP) exhibited a sensitivity of 85.7% and specificity of 96.2% with a 0.0% incidence of FN. Multimodal IONM (EMG + MEP) demonstrated a sensitivity of 100% and specificity of 96.2% with a 0.01% incidence of FN. Compared to unimodal options, multimodal IONM provides timely alerts to avoid damage to nerve roots caused by long-term stretching and compression during PELIF, increasing the detection of neurological complications. Notably, when there is an abnormal potential, we have carried out timely observation and treatment. All occurred neurological deficits were transient and recovered within a week, indicating the importance of our intraoperative monitoring.

MEP primarily reflects the function and integrity of the descending motor pathway of the cortical spinal tract. The MEP monitoring method stimulates the motor cortex of the cerebrum, recording the evoked potential response in the corresponding muscle. We conducted continuous monitoring when surgeries involve key facets, and we induced MEP to combine judgment when there was EMG activity. Our results provide evidence that inclusion of MEP significantly reduces the incidence of pure motor dysfunction compared to monitoring using EMG alone, effectively improving the sensitivity of IONM. In our study, two cases were indeterminate. It was similar to Wang’s research [25]. It is difficult to conclude that whether the MEP recovery of the two cases occurred due to intra-operative immediate measures or due to false positive. Some studies determined this indeterminate situation as true positive. However, in our opinion, it is inappropriate because many factors are able to result in the MEP change, such as blood pressure, body temperature, length of operation, anesthesia [26]. Narcotic drugs significantly interfere with this process, almost all inhaled anesthetics can easily inhibit the excitatory conduction of MEP in the motor cortex, anterior horn of the spinal cord, neuromuscular junctions, etc., resulting in decreased amplitude and prolonged latency [27]. The alarm standard set by our research included an amplitude drop greater than 80%, and some were greater than 50% [28]. Moreover, a 70% decrease in the MEP area was previously used as a criterion for warning in IONM [29]. However, which standard should be used for endoscopic lumbar inter-body fusion surgery requires further investigation.

F-EMG is less affected by anesthesia and was one of the earliest methods used for IONM during lumbar spine surgery [30, 31]. EMG monitoring continuously and dynamically reflects the state of target nerve roots during surgery. Therefore, when the nerve roots continue to be stretched, compressed, and shocked during surgery, this method provides feedback in real time to avoid neurological deficits. In our research, when the invasive surgical channel was tapped by the bone hammer, activity appeared in response to the shock of the tapping, but when the tapping stopped, the activity stopped immediately. According to our analysis, this is caused by the shock of being struck, and in this case, no new neurological deficits occurred after surgery. A total of 11 cases of explosive continuous myoelectric response were observed in this study. This activity is not the same as the regular activity caused by the shock because the duration of the activity was greater than 3 s and was irregular. There were 8 cases during nucleus pulposus resection and 7 cases during cage implantation, which may be related to stimulation of the nerve root or continuous squeezing of the nerve root. However, after making corresponding adjustments, the levels returned to normal before the end of the surgery. There were also two false negatives in our study, and based on standards from previous reports [32, 33], the following constitute false negatives: (1) complete and regular nerve root cut off, causing only a small burst of activity on the EMG or no activity; (2) severe injury of the nerve; and (3) EMG cannot immediately detect nerve damage caused by bipolar radiofrequency burning because at this time, a lot of interference waves mask the true EMG response waveform. The first and second examples of false negatives did not appear in our study, and all cases showed different levels of interference waves when using bipolar radio frequency, as well as when radio frequency was used around the nerve root. Abnormal myoelectric response waveforms can be observed in a large number of interference waves, but it is difficult to clearly determine that all cases quickly return to normal after the use of radiofrequency. In general, the EMG response is more objective and serves as a timely reminder for avoiding post-operative neurological complications caused by long-term compression and stretching of the nerve root.

SSEP tests were used to assess the spinal cord integrity of the dorsal column pathway. A change in EP could indicate an insult to the sensory pathway that results in a post-operative sensory deficit [34], but based on our data, SSEP sensitivity was very poor (25.0%), indicating that the SSEP test in PELIF procedures is not useful for indicating significant post-operative sensory deficits. Furthermore, not even the combinations of different types of IONM tests were adequate to convey high specificity to detect post-operative sensory deficits due to the mixed nerve SSEPs (i.e., posterior tibial nerve [PTN] stimulation) having little utility for monitoring individual nerve root function [35]. Moreover, if the patient has sensory deficits in the lower extremities before surgery, the SSEP waveform may appear pathological, and this is more difficult to continuously monitor during surgery. Additionally, if SSEP and EMG are monitored at the same time, SSEP interferes with EMG, making the EMG results difficult to interpret. Therefore, studies have suggested combining MEP and EMG to monitor lumbar surgery [6, 29, 36–38], but in our study, there was one case with only SSEP changing, resulting in the development of numbness and tingling in the anterior thighs. These results suggest that monitoring with SSEP better monitors the sensory function of the nervous system during surgery. Intraoperative anesthesia has a great influence on Sep. In addition, compared with inhaled anesthetics, intravenous anesthesia has less effect on SEP, and the degree of influence on SEP during operation depends on the choice and dosage of anesthesia maintenance agents and whether other drugs are used in combination. Muscle relaxant has no direct effect on SEP, on the contrary, it can inhibit the clutter produced by muscle contraction to improve the recording quality of SEP [39].

There are limitations to our study. The sample size of the current study is small. A larger sample size from multiple centers will be required in further studies. Our current results cannot be used to determine which alarm threshold is more appropriate. Relying on a 50% SSEP amplitude decrease is not restrictive, while relying on an 80% MEP amplitude decrease is restrictive. We need to investigate further and explore which alarm thresholds are most suitable for this surgery.

When performing MEP monitoring, the patient vibrates due to the current, potentially affecting the surgeon’s precision. For better intraoperative monitoring, frequent stimulation is needed to determine whether the amplitude has changed; however, we did not apply frequent stimulation because we did not want to affect the safety of the operation. Rather, we stimulated the key parts or when SSEP and EMG exhibited abnormal waveforms. Therefore, surgeons must allow neurophysiologists to perform frequent MEP trials and need to understand that many alerts may not indicate surgically produced injury.

## Conclusions

We monitored EMG during the whole procedure and monitored SSEP and MEP in key steps during the procedure. When the EMG burst time was longer than 5 s, the MEP was stimulated to allow a comprehensive judgement. It is necessary to actively communicate with the anesthesiologist and the monitoring staff before and during the operation to obtain the best monitoring effect and ensure the safety of the surgery. In conclusion, multimodal intraoperative neurophysiological monitoring has better sensitivity and specificity than unimodal intraoperative neurophysiological monitoring.

## Data Availability

All data are fully available without restriction. The database used in this study is available from the corresponding author on reasonable request.

## Abbreviations

MIS: Minimally invasive surgery
PE-TLIF: Percutaneous endoscopic transforaminal lumbar interbody fusion
IONM: Intra-operative neurophysiological monitoring
MIONM: Multimodal Intra-operative neurophysiological monitoring
OP: Operation
M: Man
F: Faman
EMG: Electromyography
SSEP: Spino-spinal evoked potentials
MEP: Spino-muscular evoked potentials
EP: Evoked Potentia
PTN: Posterior tibial nerve
TP: True-positive
TN: True-Negative
FN: False-Negative
FP: False-Positive
